# Protein-bound NAD(P)H Lifetime is Sensitive to Multiple Fates of Glucose Carbon

**DOI:** 10.1038/s41598-018-23691-x

**Published:** 2018-04-03

**Authors:** Joe T. Sharick, Peter F. Favreau, Amani A. Gillette, Sophia M. Sdao, Matthew J. Merrins, Melissa C. Skala

**Affiliations:** 1 Department of Biomedical Engineering, Vanderbilt University, Nashville, Tennessee USA; 2 Morgridge Institute for Research, Madison, WI USA; 3 Department of Biomedical Engineering, University of Wisconsin-Madison, Madison, WI USA; 4 Integrated Program in Biochemistry, University of Wisconsin-Madison, Madison, WI USA; 5 Department of Medicine, University of Wisconsin-Madison, Madison, WI USA; 6 William S. Middleton Memorial Veterans Hospital, Madison, WI USA; 7 Department of Biomolecular Chemistry, University of Wisconsin-Madison, Madison, WI USA

## Abstract

While NAD(P)H fluorescence lifetime imaging (FLIM) can detect changes in flux through the TCA cycle and electron transport chain (ETC), it remains unclear whether NAD(P)H FLIM is sensitive to other potential fates of glucose. Glucose carbon can be diverted from mitochondria by the pentose phosphate pathway (via glucose 6-phosphate dehydrogenase, G6PDH), lactate production (via lactate dehydrogenase, LDH), and rejection of carbon from the TCA cycle (via pyruvate dehydrogenase kinase, PDK), all of which can be upregulated in cancer cells. Here, we demonstrate that multiphoton NAD(P)H FLIM can be used to quantify the relative concentrations of recombinant LDH and malate dehydrogenase (MDH) in solution. In multiple epithelial cell lines, NAD(P)H FLIM was also sensitive to inhibition of LDH and PDK, as well as the directionality of LDH in cells forced to use pyruvate versus lactate as fuel sources. Among the parameters measurable by FLIM, only the lifetime of protein-bound NAD(P)H (τ_2_) was sensitive to these changes, in contrast to the optical redox ratio, mean NAD(P)H lifetime, free NAD(P)H lifetime, or the relative amount of free and protein-bound NAD(P)H. NAD(P)H τ_2_ offers the ability to non-invasively quantify diversions of carbon away from the TCA cycle/ETC, which may support mechanisms of drug resistance.

## Introduction

Reduced nicotinamide adenine dinucleotide (NADH) is a fluorescent electron donor that binds to metabolic enzymes in the cytoplasm and mitochondria. The spectral properties of NADH and its phosphorylated form, NADPH, overlap, thus their combined fluorescence is denoted NAD(P)H. NADH has a vital role in glycolysis, the tricarboxylic acid (TCA) cycle, and the electron transport chain (ETC)^1,2^. NADH and NADPH bind at least 334 known proteins in cells^3^, including enzymes that are up-regulated in cancer such as lactate dehydrogenase (LDH)^4^, pyruvate dehydrogenase (PDH)^5^, and glucose 6-phosphate dehydrogenase (G6PDH)^6^. Multiphoton fluorescence imaging of NAD(P)H is useful for probing the metabolism of living cells because it is non-damaging and does not require exogenous labeling^7^.

Flavin adenine dinucleotide (FAD) is an electron acceptor in the cell, and is also fluorescent. The optical redox ratio, defined as the fluorescence intensity of NAD(P)H to that of FAD, reflects the redox balance of the cell^8^. The optical redox ratio has been used to distinguish cancer subtypes, monitor cancer treatment response, distinguish pre-cancerous cells from normal cells, and detect stem cell differentiation^9–16^. Changes in the optical redox ratio to specific metabolic perturbations have been studied as well^9,16–19^. For example, the optical redox ratio is correlated with oxygen consumption, a key metabolic process, in breast cancer cells^18,19^. There are benefits to using a ratiometric measurement versus separate NAD(P)H and FAD intensity measurements. Both noise and spatial variation in excitation light intensity common in both NAD(P)H and FAD intensity are mitigated by using the ratio of the two fluorophores. While powerful, ratiometric intensity-based measurements, such as the optical redox ratio, can be difficult to compare between samples with different optical properties. Additionally, intensity measurements cannot distinguish free from protein-bound NAD(P)H due to their similar absorption and emission spectra.

Fluorescence lifetime imaging microscopy (FLIM) probes an additional dimension of NAD(P)H activity. The fluorescence lifetime is the time that a fluorophore remains in the excited state before returning to the ground state through photon emission. The fluorescence lifetime is sensitive to changes in the microenvironment of a fluorophore, including molecular conformation, binding, pH, temperature, and the presence of quenchers^20^. On average, the fluorescence lifetime of free NAD(P)H is distinctly shorter than that of protein-bound NAD(P)H (represented as ${\tau }_{1}$ and ${\tau }_{2}$, respectively). This difference is likely due to the degree of motion of the excited nicotinamide ring, which is more restricted when NAD(P)H is enzyme-bound than when it is free^21^. Due to these distinct lifetimes, FLIM can quantify the relative fractions of free and protein-bound NAD(P)H in the cell (represented as ${\alpha }_{1}$ and ${\alpha }_{2}$, respectively, where ${\alpha }_{1}+{\alpha }_{2}=1$)^22^. These parameters are measured by fitting a time-resolved fluorescence decay curve to a two-component exponential decay according to Equation 3 (see methods). FLIM of endogenous fluorophores including NAD(P)H has been used to study metabolism in disease pathology and progression^23^, to monitor metabolic response to therapy^24,25^, and to distinguish cancerous from normal cells and tissues^9,11,26^. FLIM of NAD(P)H has been applied to cell culture^9,27–29^, to animal models of disease progression^11,30,31^, and in pilot studies in human tissues *in vivo*^32^ and *ex vivo*^33–35^.

Many studies have shown that NAD(P)H FLIM can detect changes in flux through glycolysis, the TCA cycle and ETC^9,25,27,36–41^. However, it remains unclear whether NAD(P)H FLIM can be used to detect metabolic alterations at other key enzymatic steps that control the path of carbon from glucose uptake to ETC activity. These carbon-diverting steps include the pentose phosphate pathway (PPP) via G6PDH, lactate production via LDH, and inhibited entry into the TCA cycle via pyruvate dehydrogenase kinase (PDK) (PDK inhibits PDH activity). These pathways can be upregulated in cancer to support tumor progression and drug resistance^4–6,42,43^. Additionally, it remains unclear which NAD(P)H FLIM variables are most sensitive to these carbon-diverting steps.

In this study, we tested whether multiphoton NAD(P)H FLIM is sensitive to key enzymatic steps that control the path of carbon from glucose uptake to ETC activity. Experiments in solutions characterized NAD(P)H lifetimes under controlled titrations of two enzymes. To determine whether NAD(P)H lifetime is sensitive to diversions of carbon away from mitochondria, FLIM of NAD(P)H was performed on breast epithelial cells and pancreatic duct epithelial cells treated with metabolic inhibitors. These inhibitors include FX11, which inhibits LDH^44,45^, and dichloroacetate (DCA), which allows carbon to enter the TCA cycle by relieving PDK inhibition on PDH^46^. FX11 and DCA are under investigation as cancer therapies because LDH and PDH activities are dysregulated in cancer^4,42,44–47^. In addition, NAD(P)H FLIM was measured in cells forced to metabolize lactate or pyruvate, thus modulating carbon flux through LDH. Our results indicate that the fluorescence lifetime of protein-bound NAD(P)H is more sensitive to these key enzymatic steps than the NAD(P)H intensity, free NAD(P)H lifetime, or relative amounts of free and protein-bound NAD(P)H.

## Results

### FLIM of NAD(P)H bound to enzymes in solution

**Figure 1:**
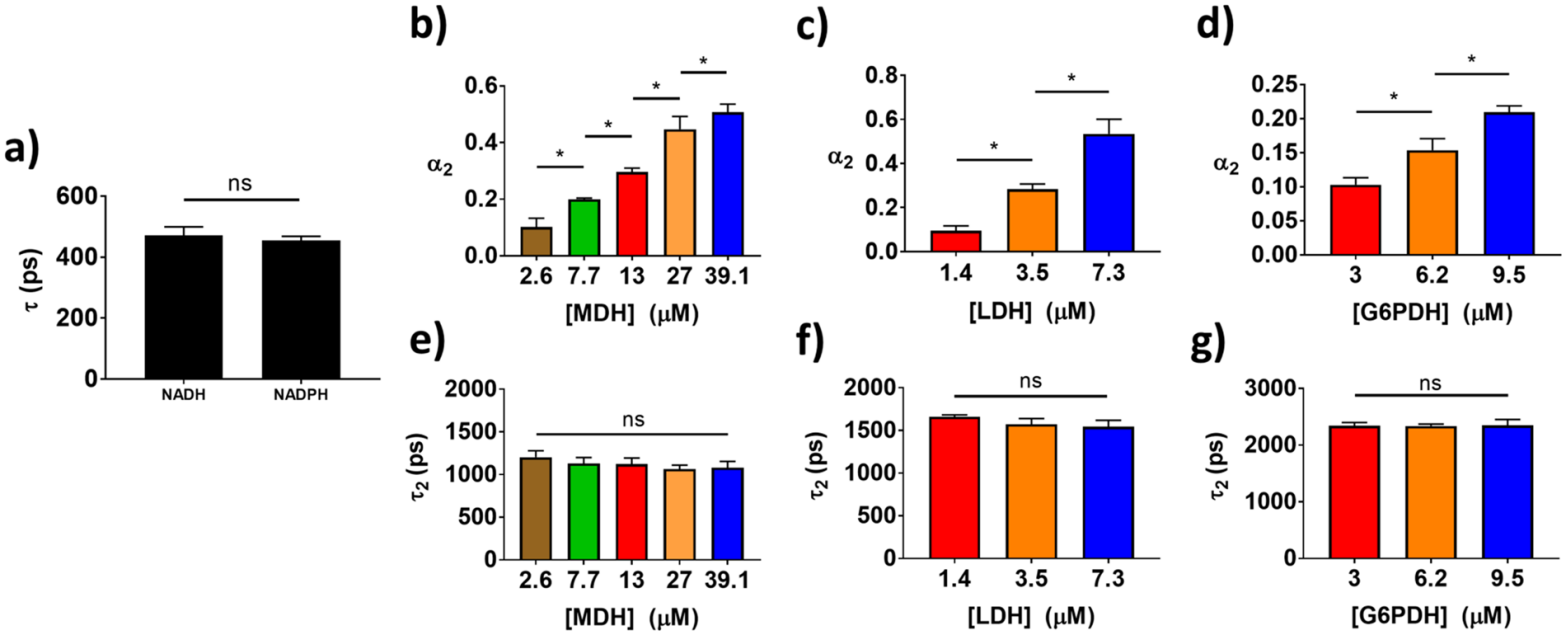
FLIM of NADH bound to metabolic enzymes in solution. (**a**) Mean and standard deviations of the fluorescence lifetime of 50 μM NADH and 50 μM NADPH from 3–5 experiments. ns = p > 0.05. (**b,c**) Mean and standard deviations of the fraction of protein-bound NADH, or ${\alpha }_{2}$, measured in mixtures of 50 μM NADH with varied concentrations of (**b**) MDH or (**c**) LDH across triplicate experiments. *p < 0.05. (**d**) Mean and standard deviations of NADPH ${\alpha }_{2}$ measured in mixtures of 50 μM NADPH with varied concentrations of G6PDH across triplicate experiments. (**e–g**) Mean and standard deviations of the protein-bound lifetime of NAD(P)H, or ${\tau }_{2}$, measured in the same solutions of NADH with MDH (**e**), NADH with LDH (**f**), and NADPH with G6PDH (**g**) across triplicate experiments.

Solutions of NAD(P)H and metabolic enzymes were generated to determine whether FLIM could accurately recover relative enzyme concentrations. First, separate solutions of 50 μM NADH and 50 μM NADPH were imaged to determine their free fluorescence lifetime. No difference in lifetime was found between the two species (Fig. 1a). Next, solutions of NADH or NADPH with varying concentrations of enzymes, MDH, LDH, and G6PDH, were generated and imaged. As expected, the fraction of protein-bound NADH, ${\alpha }_{2}$, reflected the relative concentration of malate dehydrogenase (MDH) and LDH in solution (Fig. 1b,c). NADPH ${\alpha }_{2}$ reflected the relative concentration of G6PDH in solution (Fig. 1d). The measured value for the lifetime of NADH bound to MDH or LDH (${\tau }_{2})$ was consistent between NADH-to-enzyme ratios (p > 0.05), with an average lifetime of 1.2 ns or 1.6 ns, respectively (Fig. 1e,f). Likewise, the lifetime of NADPH bound to G6PDH (${\tau }_{2}$) did not change between solutions (p > 0.05), and had an average value of about 2.5 ns (Fig. 1g). Supplementary Fig. 1 shows representative decay curves and the corresponding multi-exponential fits that were used to calculate the NAD(P)H lifetimes. Supplementary Fig. 2 shows representative images of these solutions, demonstrating their homogeneity.

**Figure 2:**
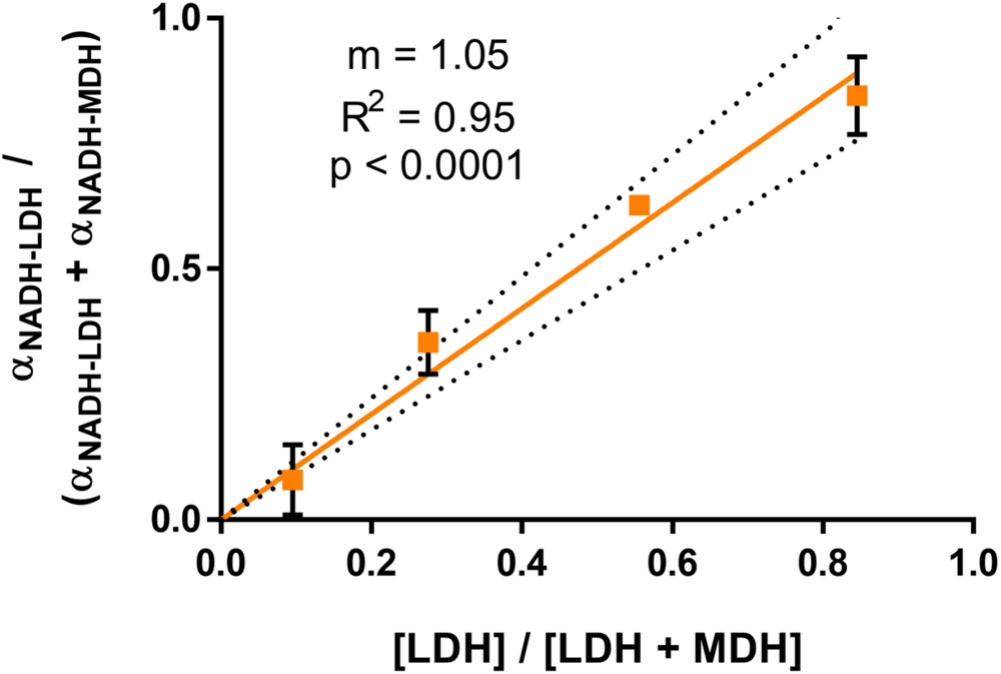
FLIM of NADH quantifies a mixture of enzymes in solution. The relationship between the relative concentration of LDH in a solution of NADH, LDH, and MDH, and the measured ${\alpha }_{NADH-LDH}$ value (Eq. 4). Error bars represent the standard deviation across triplicate experiments, and the dotted line represents the 95% confidence interval for the regression line. Error bars on one of the points are too small to display (SD =  ± 0.004). The linear regression is forced through 0,0, and the slope of the line is given as m. [LDH], concentration of LDH in the solution; [LDH + MDH], concentration of LDH plus MDH in the solution. ${\alpha }_{NADH-LDH}$ and ${\alpha }_{NADH-MDH}$ from Eq. 4.

Solutions containing a fixed concentration of 50 μM NADH and both LDH and MDH were generated to determine if FLIM could quantify the relative amounts NADH bound to two different enzymes. The FLIM-measured proportion of protein-bound NADH attributed to LDH was compared to the actual proportion of LDH added to each mixture (Fig. 2). The R^2^ was 0.95 (p < 0.0001), indicating a strong correlation between FLIM-measured enzyme proportions and the actual enzyme proportions. Table 1 shows the concentrations of enzymes used to generate the mixtures shown in Fig. 2, along with the proportion of free NADH (${\alpha }_{NADH-Free}$, Eq. 4) measured in each solution. Examples of the three-exponential fits (Eq. 4) that were used to calculate these proportions are shown in Supplementary Fig. 3.

**Table 1:** NADH-MDH-LDH solution parameters.

	Mixture 1	Mixture 2	Mixture 3	Mixture 4
[LDH] (μM)	4.9	14.2	1.4	10.0
[MDH] (μM)	13.0	2.6	13.0	8.0
[LDH]/([LDH] + [MDH])	0.27	0.85	0.10	0.56
Total Enzyme (μM)	17.9	16.8	14.4	18.0
Measured α_NADH-Free_ (%)	60.5	48.0	68.9	42.6

### Effects of inhibitors on cell enzyme activities

**Figure 3:**
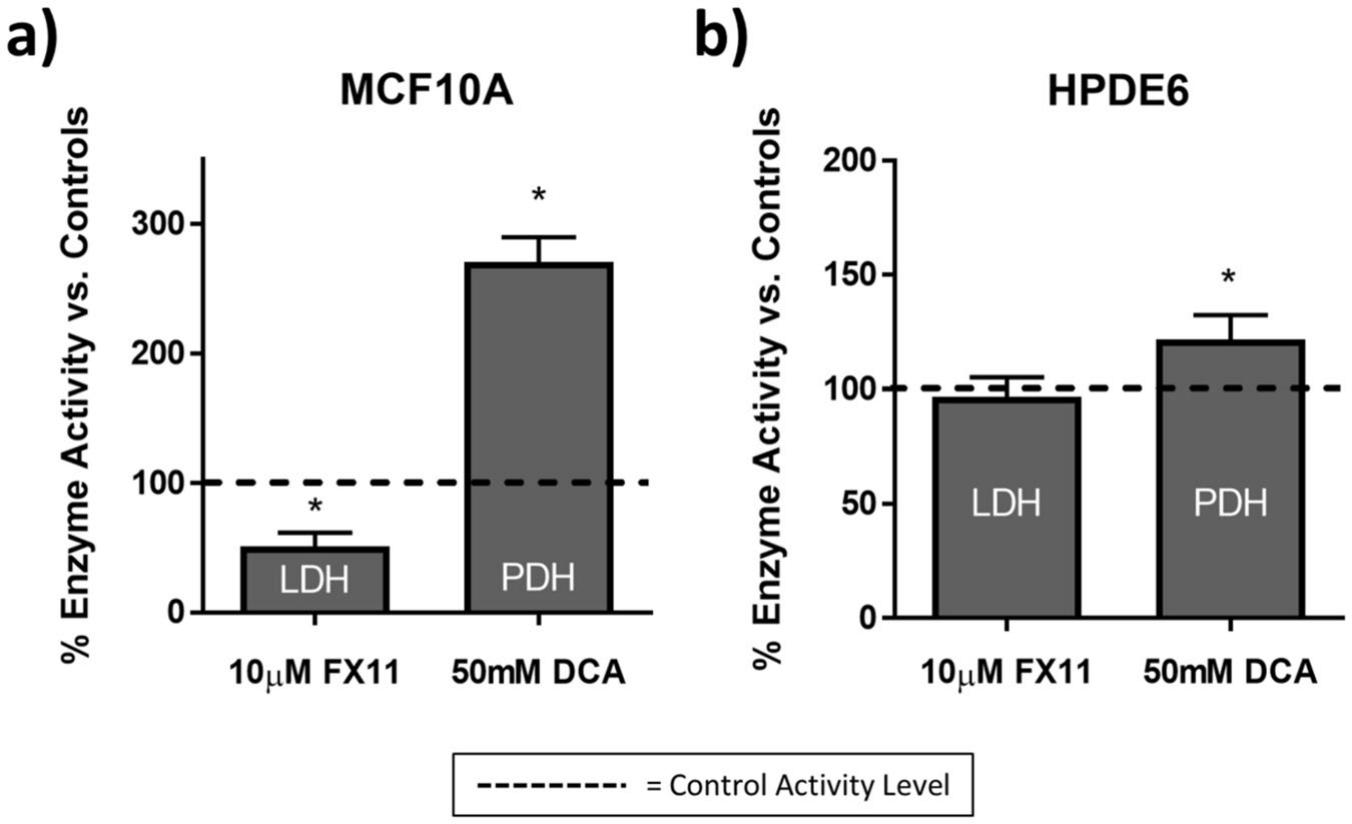
Relative activities of LDH and PDH with FX11 and DCA treatment in cells. Mean and standard deviations of the percent change in LDH and PDH activity after 48 hours of FX11 or 48 hours of DCA treatment, respectively in MCF10A cells (**a**) and HPDE6 cells (**b**) vs. vehicle. Dashed line represents enzyme activity level in vehicle-treated cells. *p < 0.05 vs. control. n = 3–5 experiments.

**Figure 4:**
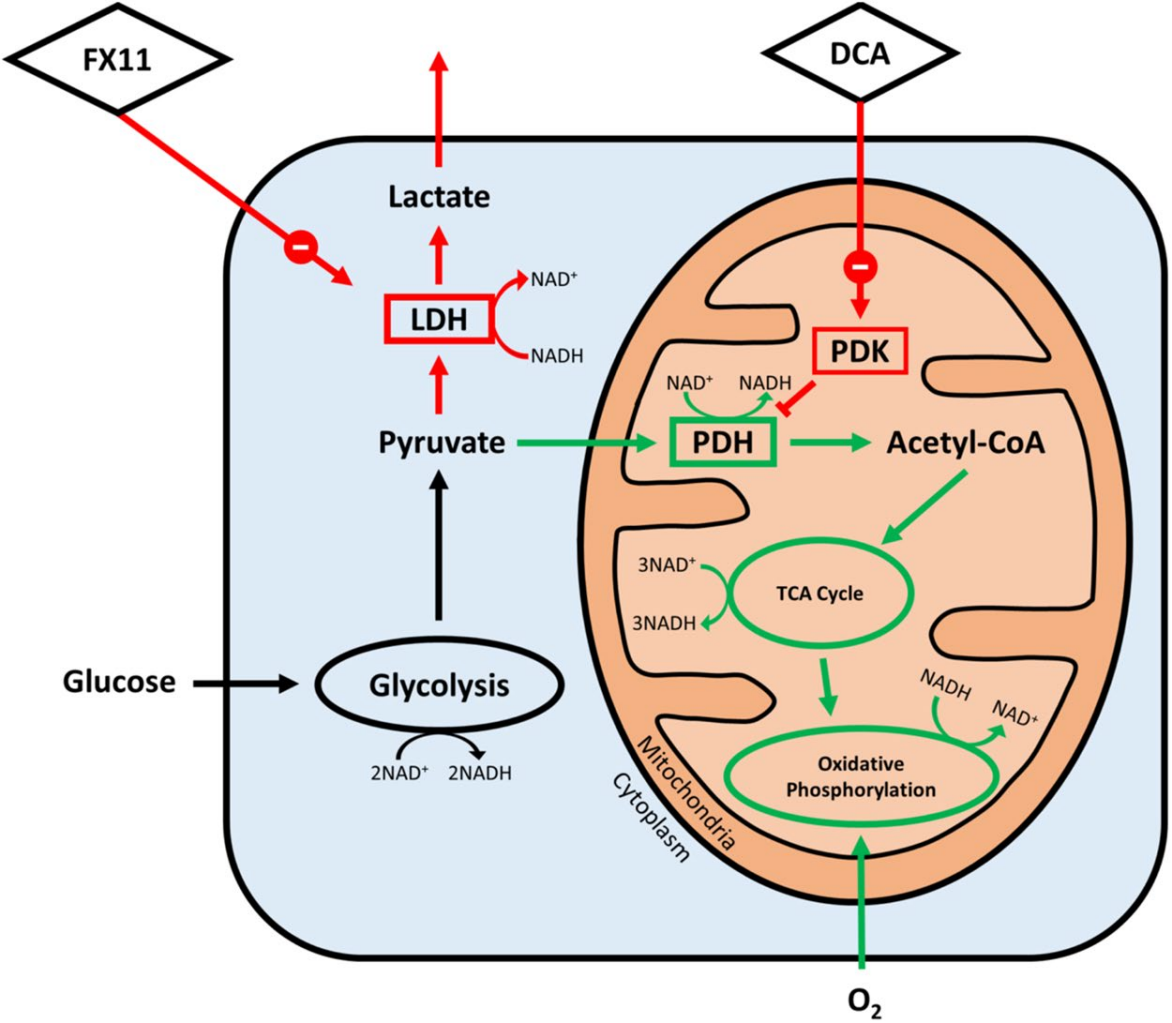
Metabolic diagram of enzyme inhibition with DCA and FX11 treatment. Summary of cellular metabolic pathways investigated using metabolic inhibitors DCA and FX11 (diamonds). Red arrows indicate a decrease in activity, while green arrows indicate an increase, and black arrows indicate no change. Enzymes are shown in boxes, enzyme-containing pathways such as glycolysis and oxidative phosphorylation are circled, while enzyme products and substrates are unboxed. Both FX11 and DCA result in an overall increase in oxygen consumption, but by acting on different enzymes. DCA inhibits PDK to increase the activity of PDH, allowing pyruvate to enter the mitochondria and fuel oxidative phosphorylation. FX11 has a similar effect, but acts by inhibiting the ability of LDH to convert pyruvate into lactate.

Standard measures of enzyme activity after FX11 treatment confirmed a significant decrease in LDH activity (p < 0.05, Fig. 3a), while DCA treatment caused a significant increase in PDH activity (p < 0.005, Fig. 3a) compared to control in MCF10A breast epithelial cells. In HPDE6 pancreatic duct epithelial cells, FX11 had no effect on LDH activity (Fig. 3b), while DCA caused a significant increase in PDH activity (p < 0.05, Fig. 3b). The absolute activity rates of LDH and PDH are shown in Supplementary Fig. 4. The absolute activity of LDH at baseline was over one order of magnitude higher than PDH in both MCF10A and HPDE6 cells. Supplementary Fig. 5 gives examples of the time-dependent enzyme reaction that was used to calculate the enzyme activity rates. The established mechanisms and targets of these metabolic inhibitors are diagrammed in Fig. 4.

### Optical redox ratio in cells with enzyme inhibitors

**Figure 5:**
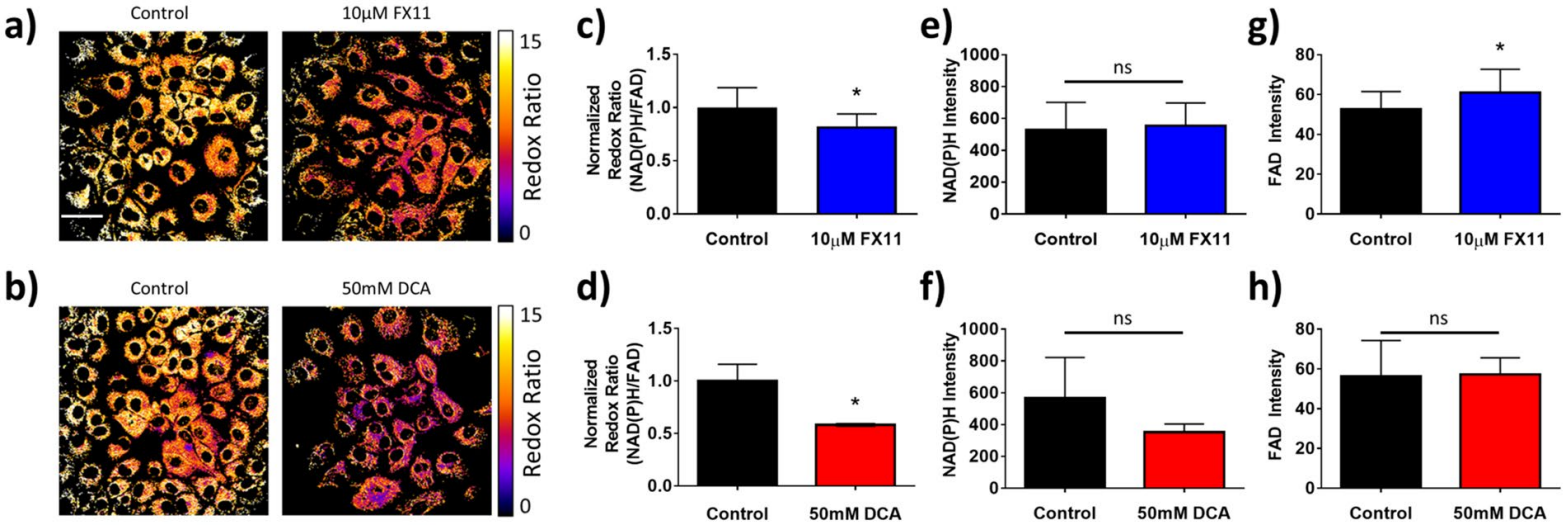
Effects of FX11 and DCA on the optical redox ratio in MCF10A cells. (**a–c**) Representative images of MCF10A cells after 48 hours of 10 μM FX11 (**a**) and 48 hours of 50 mM DCA (**b**) treatment vs. vehicle, color-coded for the redox ratio. Scale bar = 50 μm. (**c,d**) Mean and standard deviations of the normalized redox ratio in MCF10A cells after 48 hours of 10 μM FX11 (**c**) and 48 hours of 50 mM DCA (**d**) treatment vs. vehicle. *p < 0.05 vs. control. n = 3 experiments. (**e,f**) Mean and standard deviations of NAD(P)H fluorescence intensity in MCF10A cells after 48 hours of 10 μM FX11 (**e**) and 48 hours of 50 mM DCA (**f**) treatment vs. vehicle. ns = p > 0.05 vs. control. n = 3 experiments. (**g,h**) Mean and standard deviations of FAD fluorescence intensity in MCF10A cells after 48 hours of 10 μM FX11 (**g**) and 48 hours of 50 mM DCA (**h**) treatment vs. vehicle. n = 3 experiments.

Multiphoton FLIM visualizes autofluorescence changes on an individual cell level in MCF10A cells (Fig. 5a,b). No changes in cell morphology were noted with drug treatment. Both FX11 and DCA significantly (p < 0.05) decreased the optical redox ratio in MCF10A cells compared to control (Fig. 5c,d). The fluorescence intensities of NAD(P)H (Fig. 5e,f) and FAD (Fig. 5g,h) alone do not significantly change with treatment in MCF10A cells except for an increase in FAD intensity with FX11 treatment (p < 0.05).

**Figure 6:**
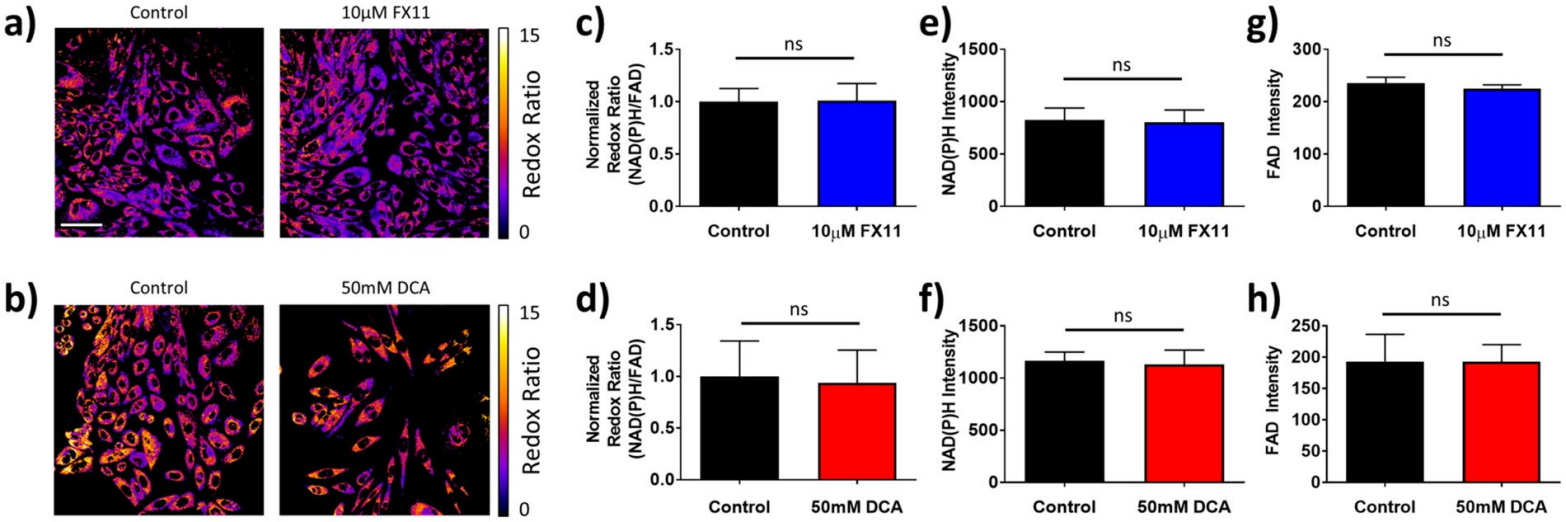
Effects of FX11 and DCA on the optical redox ratio in HPDE6 cells. (**a,b**) Representative images of HPDE6 cells after 48 hours of 10 μM FX11 (**a**) and 48 hours of 50 mM DCA (**b**) treatment vs. vehicle, color-coded for the redox ratio. Scale bar = 50 μm. (**c,d**) Mean and standard deviations of the normalized redox ratio in HPDE6 cells after 48 hours of 10 μM FX11 (**c**) and 48 hours of 50 mM DCA (**d**) treatment vs. vehicle. ns = p > 0.05 vs. control. n = 3 experiments. (**e,f**) Mean and standard deviations of NAD(P)H fluorescence intensity in HPDE6 cells after 48 hours of 10 μM FX11 (**e**) and 48 hours of 50 mM DCA (**f**) treatment vs. vehicle. n = 3 experiments. (**g,h**) Mean and standard deviations of FAD fluorescence intensity in HPDE6 cells after 48 hours of 10 μM FX11 (**g**) and 48 hours of 50 mM DCA (**h**) treatment vs. vehicle. n = 3 experiments.

No changes in cell morphology were noted with drug treatment in HPDE6 cells (Fig. 6a,b). Additionally, neither FX11 nor DCA caused significant changes to the optical redox ratio (Fig. 6c,d), NAD(P)H fluorescence intensity (Fig. 6e,f), or FAD intensity (Fig. 6g,h) in HPDE6 cells.

### NAD(P)H fluorescence lifetime in cells with enzyme inhibitors

**Figure 7:**
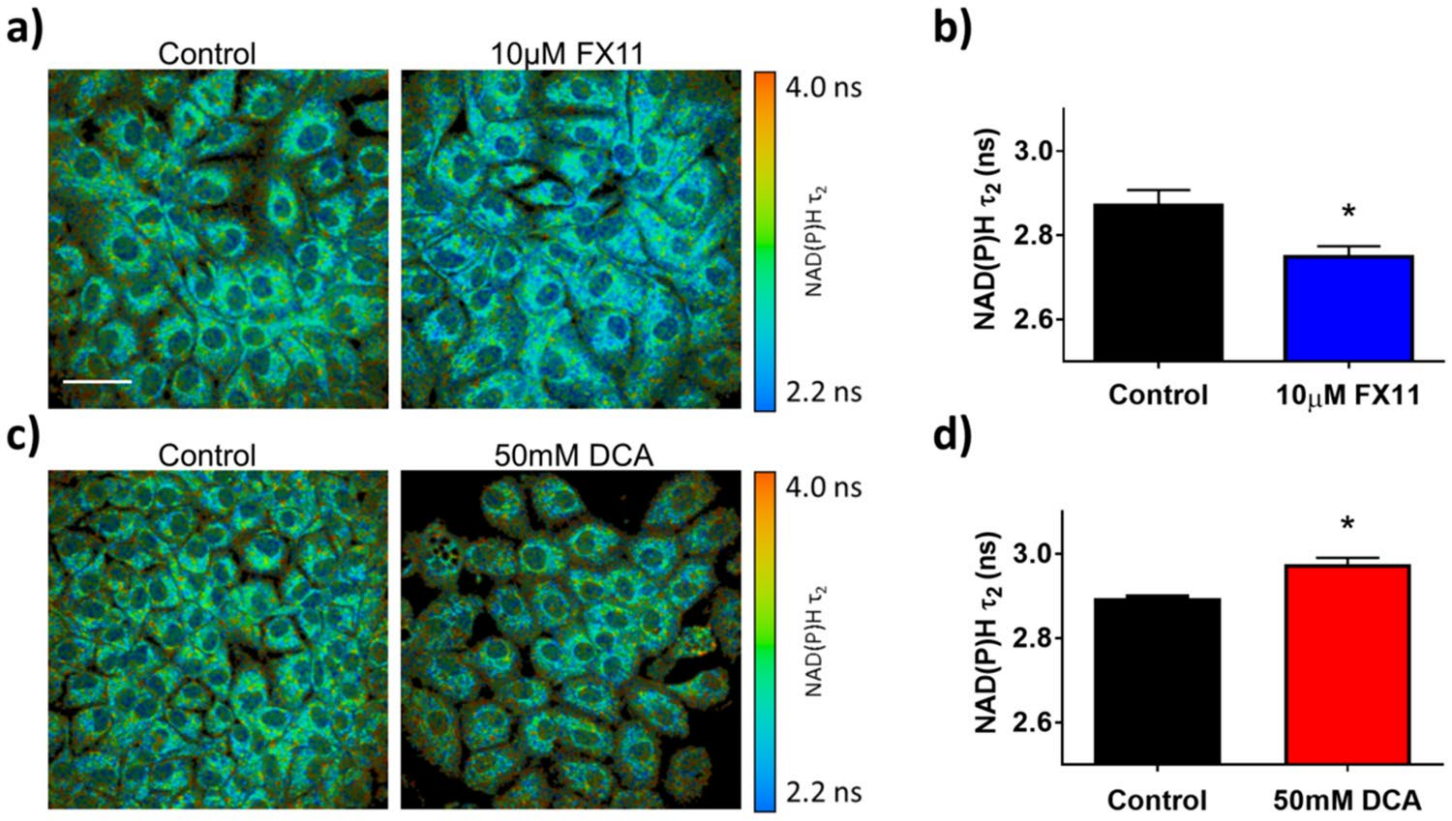
Effects of FX11 and DCA on the fluorescence lifetime of protein-bound NAD(P)H in MCF10A cells. (**a**) Representative images of MCF10A cells after 48 hours of 10 μM FX11 treatment vs. vehicle, color-coded for NAD(P)H ${\tau }_{2}$. Scale bar = 50 μm. (**b**) Mean and standard deviations of NAD(P)H ${\tau }_{2}$ in MCF10A cells after 48 hours of 10 μM FX11 treatment vs. vehicle. *p < 0.05 vs. control. n = 3 experiments. (**c**) Representative images of MCF10A cells after 48 hours of 50 mM DCA treatment vs. vehicle color-coded for NAD(P)H ${\tau }_{2}$. (**d**) Mean and standard deviations of NAD(P)H ${\tau }_{2}$ in MCF10A cells after 48 hours of 50 mM DCA treatment vs. vehicle. n = 3 experiments.

**Figure 8:**
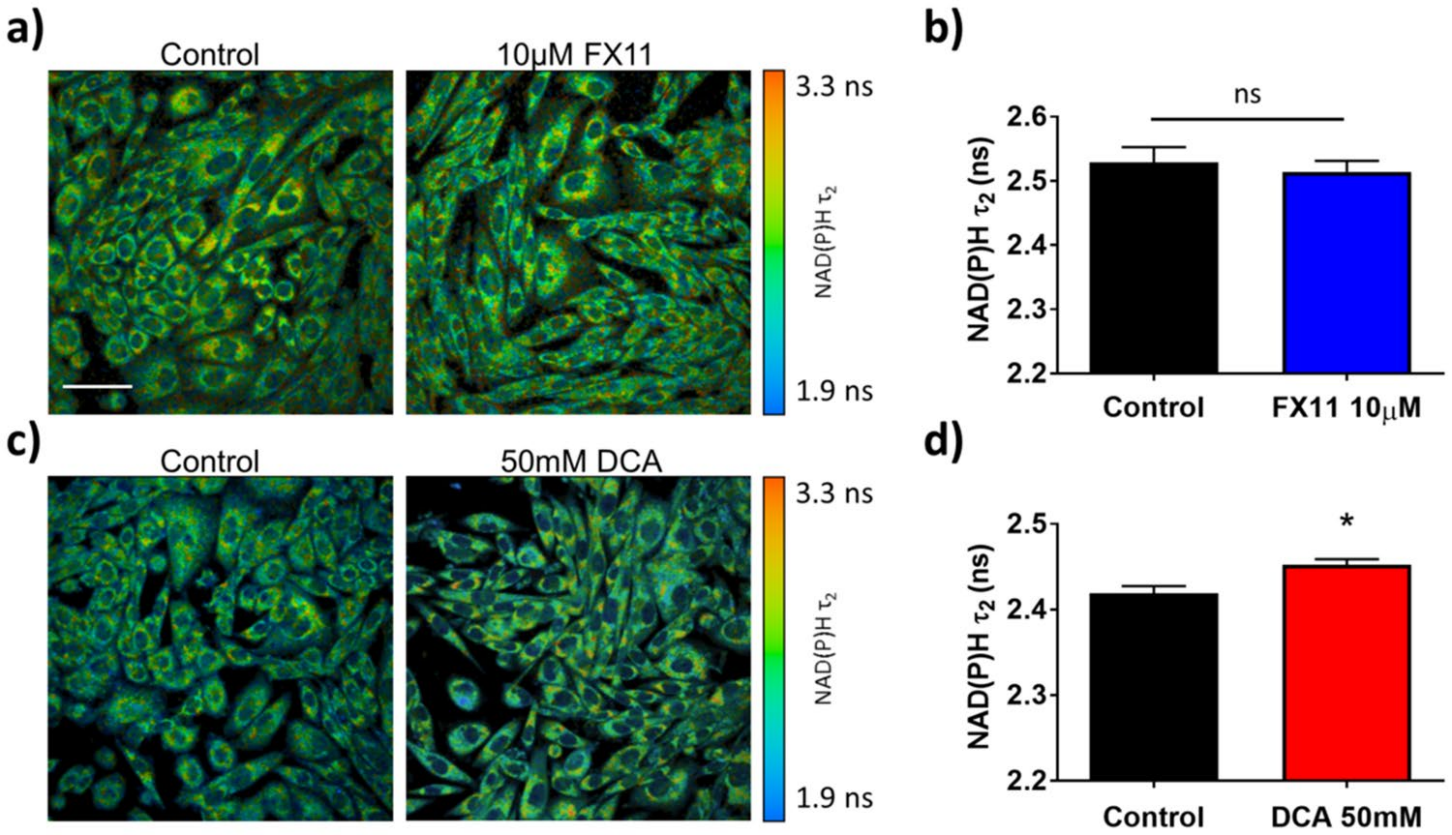
Effects of FX11 and DCA on the fluorescence lifetime of protein-bound NAD(P)H in HPDE6 cells. (**a**) Representative images of HPDE6 cells after 48 hours of 10 μM FX11 treatment vs. vehicle, color-coded for NAD(P)H ${\tau }_{2}$. Scale bar = 50 μm. (**b**) Mean and standard deviations of NAD(P)H ${\tau }_{2}$ in HPDE6 cells after 48 hours of 10 μM FX11 treatment vs. vehicle. *p < 0.05 vs. control. n = 4 experiments. (**c**) Representative images of HPDE6 cells after 48 hours of 50 mM DCA treatment vs. vehicle color-coded for NAD(P)H ${\tau }_{2}$. (**d**) Mean and standard deviations of NAD(P)H ${\tau }_{2}$ in HPDE6 cells after 48 hours of 50 mM DCA treatment vs. vehicle. n = 4 experiments.

FX11 treatment significantly decreased NAD(P)H ${\tau }_{2}$ in MCF10A cells (p < 0.0001, Fig. 7a,b). Conversely, DCA treatment significantly increased NAD(P)H ${\tau }_{2}$ (p < 0.01, Fig. 7c,d). FX11 treatment did not have a significant effect on NAD(P)H ${\tau }_{2}$ in HPDE6 cells (Fig. 8a,b). DCA treatment significantly increased NAD(P)H ${\tau }_{2}$ in HPDE6 cells (p < 0.05, Fig. 8c,d).

**Figure 9:**
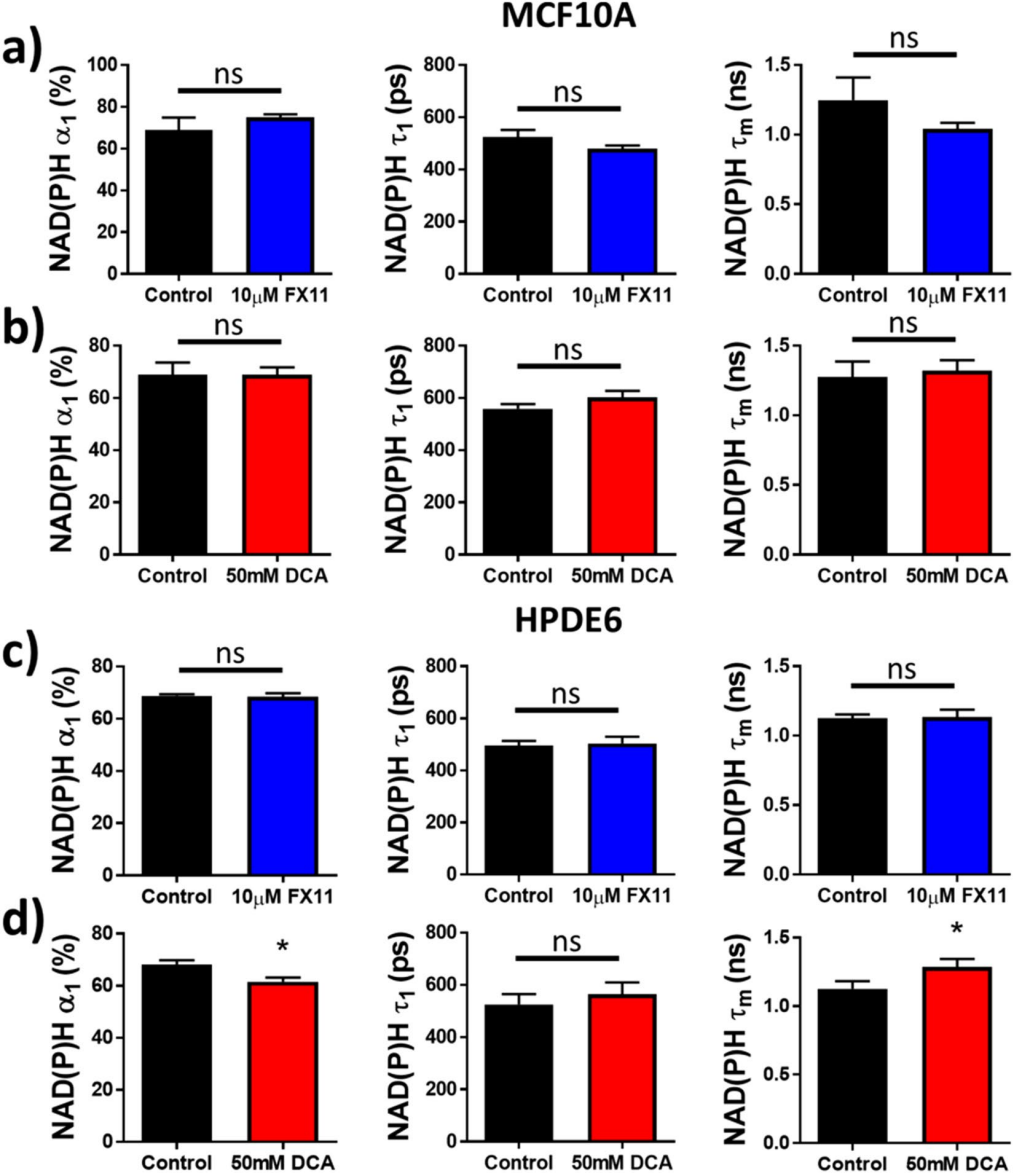
Sensitivity of additional NAD(P)H fluorescence lifetime components to FX11 and DCA treatment in cells. (**a**) Mean and standard deviations of NAD(P)H ${\alpha }_{1}$, ${\tau }_{1}$, and ${\tau }_{m}$ in MCF10A cells after 48 hours of 10 μM FX11 treatment vs. vehicle. (**b**) Mean and standard deviations of NAD(P)H ${\alpha }_{1}$, ${\tau }_{1}$, and ${\tau }_{m}$ in MCF10A cells after 48 hours of 50 mM DCA treatment vs. vehicle. (**c**) Mean and standard deviations of NAD(P)H ${\alpha }_{1}$, ${\tau }_{1}$, and ${\tau }_{m}$ in HPDE6 cells after 48 hours of 10 μM FX11 treatment vs. vehicle. (**d**) Mean and standard deviations of NAD(P)H ${\alpha }_{1}$, ${\tau }_{1}$, and ${\tau }_{m}$ in HPDE6 cells after 48 hours of 50 mM DCA treatment vs. vehicle. n = 3–4 experiments each.

The effects of these inhibitors on the additional NAD(P)H lifetime parameters,$\,{\alpha }_{1}$, ${\tau }_{1}$, or ${\tau }_{m}$, were analyzed. Neither inhibitor had a significant effect on NAD(P)H ${\alpha }_{1}$, ${\tau }_{1}$, or ${\tau }_{m}$ in MCF10A cells (Fig. 9a,b). Most of these lifetime parameters did not change with inhibitor treatment in HPDE6 cells, other than a significant decrease in the proportion of free NAD(P)H and a significant increase in NAD(P)H ${\tau }_{m}$ with DCA treatment (Fig. 9c,d). Unlike NAD(P)H ${\tau }_{2}$, these additional NAD(P)H lifetime parameters do not consistently reflect changes in enzyme activity across both cell types and inhibitors. Representative decay curves and the corresponding multi-exponential fits that were used to calculate these NAD(P)H lifetime parameters are in Supplementary Fig. 6.

### Autofluorescence in cells with varying fuel sources

**Figure 10:**
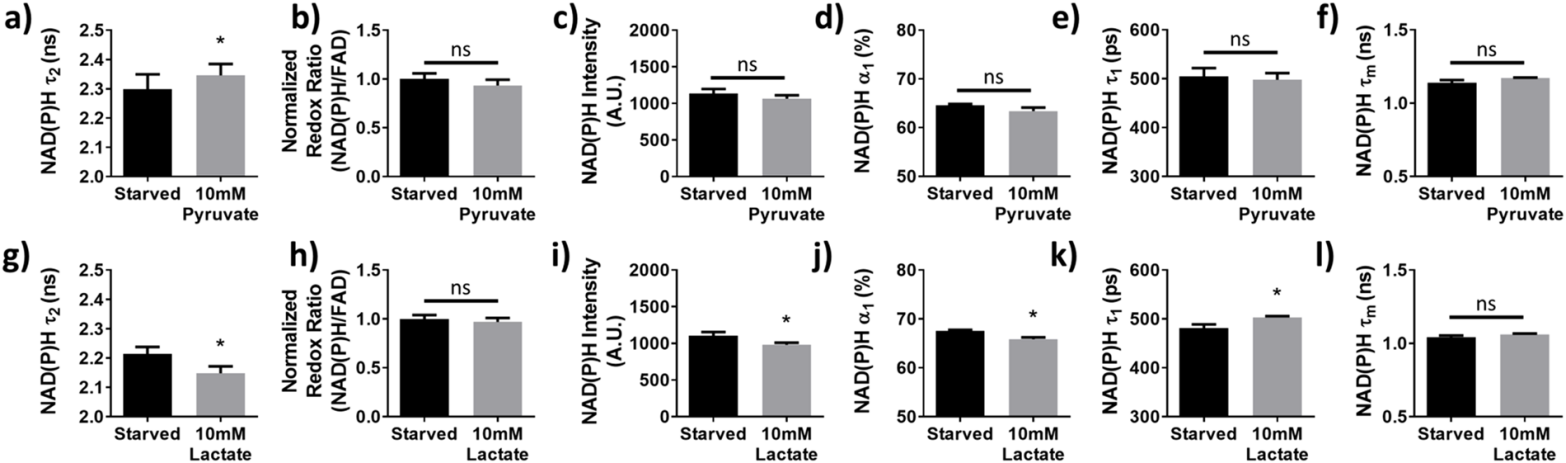
NAD(P)H ${\tau }_{2}$ is sensitive to cellular fuel source. (**a–f**) Mean and standard deviations of NAD(P)H ${\tau }_{2}$ (**a**), optical redox ratio (**b**), NAD(P)H intensity (**c**), NAD(P)H ${\alpha }_{1}$ (**d**), NAD(P)H ${\tau }_{1}$ (**e**), and NAD(P)H ${\tau }_{m}$ (**f**) of fuel-starved MCF10A cells before and after addition of 10 mM sodium pyruvate. (**g–l**) Mean and standard deviations of NAD(P)H ${\tau }_{2}$ (**g**), optical redox ratio (**h**), NAD(P)H intensity (**i**), NAD(P)H ${\alpha }_{1}$ (**j**), NAD(P)H ${\tau }_{1}$ (**k**), and NAD(P)H ${\tau }_{m}$ (**l**) of fuel-starved MCF10A cells before and after addition of 10 mM sodium lactate. ns = p > 0.05 vs. control. *p < 0.05 vs. control. n = 3 experiments each.

Like enzyme inhibitors, the fuel source available to the cell also alters carbon flux. Therefore, the effect of fuel source on autofluorescence in MCF10A cells was tested to complement the above enzyme inhibitor experiments. Specifically, the diversion of carbon from mitochondria can be modulated by forcing starved cells to use lactate versus pyruvate as a primary fuel source (Fig. 4). NAD(P)H ${\tau }_{2}\,$increased significantly after MCF10A cells were supplied with an excess of pyruvate (p < 0.05, Fig. 10a), while the optical redox ratio, NAD(P)H fluorescence intensity,$\,{\alpha }_{1}$, ${\tau }_{1}$, and ${\tau }_{m}\,$did not change (Fig. 10b–f). When the cells were supplied with excess lactate, NAD(P)H ${\tau }_{2}\,$decreased (p < 0.05, Fig. 10g), while the optical redox ratio and NAD(P)H ${\tau }_{m}$ remained unchanged (Fig. 10h,l). The NAD(P)H fluorescence intensity and ${\alpha }_{1}$ decreased slightly with lactate (p < 0.05, Fig. 10I,j), while ${\tau }_{1}$ increased (p < 0.05, Fig. 10k). NAD(P)H ${\tau }_{2}\,$more robustly distinguishes the use of pyruvate or lactate as fuel sources in cells than the optical redox ratio, NAD(P)H ${\tau }_{1}$, ${\alpha }_{1},\,{\tau }_{m}$ or intensity. Representative decay curves and the corresponding multi-exponential fits that were used to calculate these NAD(P)H lifetime parameters can be found in Supplementary Fig. 7.

## Discussion

FLIM of NAD(P)H can non-destructively measure changes in cellular metabolism, but these changes are difficult to interpret. In this study, we used NAD(P)H-enzyme solutions, metabolic inhibitors in cells, and alternate fuel sources for cells to determine if NAD(P)H FLIM is sensitive to key enzymatic steps that control the path of carbon from glucose uptake to ETC activity. This information will aid in the interpretation of changes in NAD(P)H lifetimes.

The measured lifetimes of NADH bound to LDH and MDH in solution were distinct from each other (Fig. 1) and only slightly different than previously reported values^22,36^. Deviations from previous values are likely due to differences in buffer conditions, different fluorescence emission filter wavelengths, different numbers of exponential decays used for fitting, or differences in temporal resolution between previous single-point lifetime measurements and our FLIM measurements. Previous studies show that LDH and MDH have distinct effects on the fluorescence spectrum of NADH, which suggests unique molecular conformations and/or environments^48^. We also found that the fluorescence lifetime of NADPH bound to the NADPH-dependent enzyme G6PDH was independent of enzyme concentration, and was distinct from the lifetimes of NADH bound to MDH or LDH. This agrees with the previous report that FLIM can be used to distinguish protein-bound NADH from protein-bound NADPH^49^. The consistent protein-bound NADH lifetime across enzyme concentration within a single-enzyme solution (Fig. 1f–h) was expected because fluorescence lifetimes are independent of the fluorophore concentration^20,50^. Small differences in protein-bound NAD(P)H lifetime between enzyme concentrations within a single enzyme solution are likely due to time-correlated single photon counting (TCSPC) noise and fitting error^51^. MDH, LDH, and G6PDH were chosen for this study because they are readily available in isolated form, can be dissolved *in vitro* in the same buffers as NADH, and have well-characterized binding affinities for NADH^52–54^. Additionally, these experiments tested whether an enzyme in the TCA cycle (MDH) could be distinguished from key enzymes that modify the flow of carbon into the TCA cycle (LDH and G6PDH).

This is the first study to demonstrate that NAD(P)H ${\tau }_{2}$ can distinguish NADH bound to two different metabolic enzymes in a single solution, and thus quantify changes in the relative activities of two enzymes. We found a strong linear correlation between the relative amounts of LDH and MDH added to a solution of NADH, and the measured values of ${\alpha }_{NADH-LDH}$ and ${\alpha }_{NADH-MDH}$ (Fig. 2). Without further characterization, we cannot obtain the absolute ratio of NADH molecules bound to LDH vs. MDH using FLIM, but these results do indicate that $\alpha$ values were sensitive to changes in this ratio. While it is doubtful that FLIM can distinguish all 334 known binding partners of NAD(P)H in cells^3^, the summation of protein-bound NAD(P)H lifetimes within distinct metabolic pathways may differ. Thus, changes in protein-bound NAD(P)H lifetimes could be related back to groups of metabolic reactions, including those that divert carbon away from the mitochondria.

Decreases in the optical redox ratio with both DCA and FX11 treatment in MCF10A cells (Fig. 5) agree with previous reports that these inhibitors increase oxygen consumption in other cell types^18,19,44,45^. However, the redox ratio did not distinguish the different mechanisms of DCA and FX11 treatment in MCF10A cells. FX11 has been shown to increase the NADH/NAD^+^ ratio in human lymphoma cells^44^, but an increase in NAD(P)H intensity was not observed in our studies. An increase in the NADH/NAD^+^ ratio does not necessarily result in a significant increase in NAD(P)H fluorescence intensity, because the total NAD pool may have decreased following FX11 treatment. Additionally, the effects of FX11 on NAD(P)H levels in MCF10A and HPDE6 cells have not been previously reported, and could differ from the effects on lymphoma cells. FX11 has also been shown to reduce the mitochondrial membrane potential of lymphoma cells^44^, which could affect NAD(P)H FLIM values^20^.

There was no change in the optical redox ratio for FX11 treatment in HPDE6 cells (Fig. 6c), which agrees with independent measurements of sustained LDH activity with FX11 treatment in HPDE6 cells (Fig. 3b). However, there was no change in the optical redox ratio with DCA treatment (Fig. 6d), which does not agree with independent measurements of significant changes in PDH activity with DCA treatment in HPDE6 cells (Fig. 3b). Like the MCF10A results, the insensitivity of the optical redox ratio to DCA treatment in HPDE6 cells suggests that the optical redox ratio is not robustly sensitive to changes in the activity of enzymes such as PDH and LDH, which control the path of carbon from glucose uptake to the TCA cycle. The optical redox ratio is affected by changes in the concentrations of NAD(P)H and FAD, and by changes in the lifetimes of NAD(P)H and FAD (due to differences in quantum yield^20,21,55^).

Unlike the optical redox ratio, NAD(P)H ${\tau }_{2}\,$distinguished between DCA and FX11 treatment in MCF10A cells (Fig. 7). This was expected because these inhibitors altered the activity of two different NADH-binding enzymes that influence the fate of cellular carbon (PDH and LDH) (Fig. 3a). We speculate that FX11 decreased ${\tau }_{2}$ because the lifetime of NADH bound to LDH is longer than the overall NAD(P)H ${\tau }_{2}$ (FX11 inhibits NADH-LDH binding). Similarly, we speculate that DCA increased ${\tau }_{2}$ because the lifetime of NAD(P)H bound to PDH is much longer than the overall NAD(P)H ${\tau }_{2}$ (DCA activates NADH-PDH binding). However, future work is needed to interpret the direction of these changes. While NADH bound to LDH in solution was found to have a much shorter lifetime (~1.6 ns, Fig. 1g) than cellular NAD(P)H ${\tau }_{2}$ (~2.4–2.9 ns, Figs 7 and 8b,d), it is likely that the molecular environment of the cell cytoplasm (pO_2_, pH, viscosity) significantly alters the lifetime of NADH bound to LDH^20^. LDH inhibition and PDH activation cause opposite changes in NAD(P)H ${\tau }_{2}$, but both promote downstream TCA cycle and ETC activation. This suggests that the change in ${\tau }_{2}$ is due to distinct shifts in the activities of NAD(P)H-binding enzymes that control the flow of carbon to the TCA cycle, in addition to downstream activation of the TCA cycle and ETC.

FX11 and DCA treatment in HPDE6 cells provide further evidence that NAD(P)H ${\tau }_{2}$ is sensitive to changes in the activities NAD(P)H-binding enzymes that control the fate of carbon (Fig. 8). FX11 did not affect the activity of its target enzyme, LDH (Fig. 3b). There was also no change in NAD(P)H ${\tau }_{2}$ in HPDE6 cells treated with FX11 (Fig. 8b), which provides a negative control consistent with no change in LDH activity (Fig. 3b). Furthermore, DCA increased PDH activity (Fig. 3), and caused a significant increase in NAD(P)H ${\tau }_{2}$ in both cell types (Figs 7 and 8d). Other components of the NAD(P)H fluorescence lifetime (NAD(P)H intensity, ${\alpha }_{1}$, ${\tau }_{1}$, and ${\tau }_{m}$) were not consistently sensitive to changes in metabolic enzyme activities caused by inhibitors across both cell types (Fig. 9). This suggests that NAD(P)H ${\tau }_{2}\,$is more sensitive to the NADH-binding activity of LDH and PDH than other NAD(P)H fluorescence intensity and lifetime parameters.

Finally, we tested whether NAD(P)H ${\tau }_{2}\,$is sensitive to the fuel source of the cell. Like the enzyme inhibitor experiments, feeding starved cells pyruvate versus lactate can also alter the flow of cellular carbon into the mitochondria. For example, LDH is more active with lactate versus pyruvate as a fuel source because LDH must convert lactate to pyruvate. The protein-bound lifetime of NAD(P)H increased when starved cells were given pyruvate, but decreased when starved cells were given lactate (Fig. 10). Note that the baseline conditions for the enzyme inhibitor experiments (non-starved cells) are different from these fuel source experiments (starved cells). Thus, changes in enzyme activities in the two conditions are not directly comparable. For example, adding lactate to starved cells would not reduce glycolysis because glycolysis is already at a minimum. All other autofluorescence measurements including the optical redox ratio and NAD(P)H ${\tau }_{m}$ either showed no change with lactate or pyruvate as a fuel source, or only changed with lactate. Further work is needed to interpret changes in NAD(P)H ${\alpha }_{1}$, ${\tau }_{1}$ and intensity with lactate, but could be due to changes in quenchers such as H^+^ and pO_2_^20^. While NAD(P)H ${\tau }_{1}$ increased significantly with the addition of lactate, the relatively small magnitude of the change combined with a significant decrease in its fractional contribution (${\alpha }_{1}$) to the overall fluorescence signal resulted in a non-significant increase in NAD(P)H ${\tau }_{m}$. Overall, Fig. 10 supports the conclusion that NAD(P)H ${\tau }_{2}$ is more sensitive to enzymes that control the flow of carbon into the mitochondria, compared to other NAD(P)H fluorescence intensity and lifetime parameters. Forcing starved cells to use lactate versus pyruvate as fuel caused opposite changes in NAD(P)H ${\tau }_{2}$, like LDH inhibition and PDH activation. This is further evidence that changes in ${\tau }_{2}$ are not simply due to downstream TCA cycle and ETC activation.

There are additional methods to analyze TCSPC FLIM data, such as phasor analysis^56^. For this study, we chose to fit our fluorescence decay data to the sum of exponentials. There are limitations to this method, such as the selection of the number of fit components and computational time. While the fit-free phasor approach eliminates the need for *a priori* knowledge of the number of lifetime components and simplifies FLIM analysis, we were specifically interested in measuring small changes in NAD(P)H ${\tau }_{2}$, which is not easily quantified from phasor plots of TCSPC data.

This study demonstrates that the fluorescence lifetime of protein-bound NAD(P)H is sensitive to alternative fates of glucose carbon before entry into the TCA cycle. While the optical redox ratio, mean NAD(P)H lifetime, free NAD(P)H lifetime, or relative amounts of free and protein-bound NAD(P)H can provide valuable information on changes in metabolic cofactors in the cell, NAD(P)H ${\tau }_{2}$ is most useful to quantify changes in the activity of enzymes that control the path of carbon into the TCA cycle. This is likely because NAD(P)H has unique lifetimes when bound to different enzymes^22,36,50^ (Fig. 1). The results of this study confirm that a shift in the fluorescence lifetime of NAD(P)H cannot simply be interpreted as a change in glycolysis or oxidative phosphorylation rate, because pathways that control other fates of carbon must also be incorporated.

Currently, photon counts and acquisition time limits the discrimination of multiple protein-bound lifetimes, and thus most analysis of NAD(P)H lifetime in cells is performed using a two-exponential decay. More efficient photon collection methods are under development^51^ that will enable higher-order exponential fits. Future work is also required to further interpret changes in protein-bound NAD(P)H lifetimes in cells. Overall, a better understanding of NAD(P)H FLIM signals enables unique, non-destructive insights into cellular metabolism.

## Materials and Methods

### Fluorescence lifetime imaging

Fluorescence intensity and lifetime images were acquired using a custom-built multiphoton fluorescence lifetime system (Bruker Fluorescence Microscopy, Middleton, WI), with a 40× oil-immersion objective (1.3 NA, Nikon, Tokyo, Japan) and an inverted microscope using epifluorescence illumination (TiE, Nikon). A titanium:sapphire laser (Chameleon Ultra II, Coherent, Santa Clara, CA) was tuned to 750 nm for two-photon excitation of NAD(P)H and tuned to 890 nm for two-photon excitation of FAD. A 440/80 nm bandpass filter was used to collect NAD(P)H fluorescence emission, and a 550/100 nm filter was used to collect FAD emission. A pixel dwell time of 4.8 μs collected 256 × 256 pixel images over a 270 μm × 270 μm field of view, with a total integration time of 60 seconds for cells and 120 seconds for solutions. A GaAsP PMT (H7422P-40, Hamamatsu Photonics, Hamamatsu, Japan) detected emitted photons. TCSPC electronics (SPC-150, Becker & Hickl, Berlin, Germany) were used to acquire fluorescence decay curves with 256 time bins. The second harmonic generated signal from urea crystals at 900 nm excitation was used to measure the instrument response function, which had a full width at half maximum of 220 ps. A fluorescent bead (Polysciences Inc., Warrington, PA) was imaged daily as a fluorescence lifetime validation. The single-component lifetime of the bead was stable (2.13 ± 0.03 ns, n = 16), and consistent with published values^11,24,27,30^.

### Enzyme solutions

Tris-buffered saline (diH_2_0, 50 mM Tris, 150 mM NaCl) at pH 7.6 and 21 °C was used as the solvent for all solutions. LDH from porcine heart (#L7525, Sigma) or MDH from porcine heart (#M1567, Sigma) were mixed with 50 μM NADH (#43420, Sigma) to generate solutions with distinct free-to-bound ratios of NADH. G6PDH from *S*. *cerevisiae* (#G6378, Sigma) was mixed with 50 μM NADPH (#N7505) in a similar manner. Previous reports indicate that self-quenching of NADH occurs above 250 mg/L (~375 μM)^57^ in Tris buffer. In our solution studies, 50 μM was used to minimize self-quenching. Desired concentrations of enzyme were calculated using the following equation to generate solutions with a range of free-to-bound NAD(P)H ratios:

1 $$[{\rm{Enzyme}}]=\frac{[NAD(P)H]-({\alpha }_{1})}{F\ast S}$$

Here, ${\alpha }_{1}$ represents the desired fraction of unbound NAD(P)H, *S* represents the integer number of binding sites per enzyme molecule, and *F* is the fraction of enzyme binding sites to be occupied, calculated using the following equation:

2 $$F=\frac{{\alpha }_{1}\ast [NAD(P)H]}{{\alpha }_{1}\ast [NAD(P)H]+{K}_{D}}$$

Published values for the dissociation constants (K_D_) of NAD(P)H to LDH, MDH, and G6PDH were used for these calculations, in addition to the number of NADH binding sites per enzyme^52–54,58,59^. Solutions of only 50 μM NADH or NADPH were also imaged. Four mixtures of 50 μM NADH with both LDH and MDH were generated with varying amounts of the two enzymes as described in Table 1. Enzyme concentrations in this experiment were chosen that gave a wide range of LDH to MDH ratios. Following mixing, a 100 μl droplet of each solution was placed in a separate 35 mm glass-bottom imaging dish, and a glass coverslip was placed over each droplet to reduce evaporation. Solutions were imaged at room temperature.

### Enzyme solution image analysis

A histogram of photon counts per temporal bin, or decay curve, was constructed for each image by binning all pixels together to increase photon counts for improved fitting accuracy (SNR > 3000). This decay curve was deconvolved with the instrument response function using SPCImage. For solutions with one enzyme, decay curves were fit to a two-component exponential decay according to Equation 3, where $I(t)$ represents the fluorescence intensity measured at time t after the laser pulse, and C represents a constant level of background light^20,60^.

3 $$I(t)={\alpha }_{1}ex{p}^{-t/{\tau }_{1}}+{\alpha }_{2}ex{p}^{-t/{\tau }_{2}}+C$$

A two-exponential model was chosen because the chi-squared goodness of fit value did not improve for a three-exponential model versus a two-exponential model. ${\tau }_{1}$ was fixed at 450 ps, the measured lifetime value of a pure NADH solution. For solutions with two enzymes, Equation 4 was used to fit decay curves to a three-exponential decay, with ${\tau }_{1}$ fixed at 450 ps (to represent free NADH), ${\tau }_{2}$ fixed at 1.2 ns (to represent NADH bound to MDH, or NADH-MDH) and ${\tau }_{3}$ was fixed at 1.6 ns (to represent NADH bound to LDH, or NADH-LDH).

4 $$I(t)={\alpha }_{NADH-Free}ex{p}^{-t/450ps}+{\alpha }_{NADH-MDH}ex{p}^{-t/1.2ns}+{\alpha }_{NADH-LDH}ex{p}^{-t/1.6ns}+C$$

The lifetime values of NADH used in our three-component fit (Eq. 4) were fixed based on our previous measurements in individual NADH-enzyme solutions (Fig. 1). Fixing these values reduces free fitting parameters and significantly improves the accuracy of their relative contributions^51^. The relationship between actual [LDH]/[LDH + MDH] and measured ${\alpha }_{NADH-LDH}$/(${\alpha }_{NADH-LDH}$ + ${\alpha }_{NADH-MDH}$) was calculated with a regression line with y-intercept fixed at 0. A 95% confidence interval for this line was calculated, along with its coefficient of determination and p-value.

### Cell culture, metabolic inhibition, and imaging

The MCF10A noncancerous mammary epithelial cell line (36 y.o. Caucasian female) was obtained from the American Type Culture Collection (#CRL-10317, Mannasas, VA) and grown in DMEM/F-12 (#11330, Gibco, Gaithersburg, MD) supplemented with 5% horse serum (#16050, Gibco), 20 ng/mL EGF (#AF-100-15, Peprotech, Rock Hill, NJ), 0.5 μg/mL hydrocortisone (#H0888, Sigma, St. Louis, MO), 100 ng/mL cholera toxin (#C8052, Sigma), 10 μg/mL insulin (#I1882, Sigma), and 1% penicillin/streptomycin (#15070, Gibco). The HPDE6 human pancreatic duct epithelial cells were obtained from ABM (#T0005, Richmond, BC, Canada) and grown in DMEM (#11965, Gibco) supplemented with 10% fetal bovine serum (#TMS-013-B, EMD Millipore) and 1% penicillin/streptomycin. For imaging, 1 × 10^5^ cells were seeded 24 hours prior to drug treatment in 35 mm glass-bottom dishes (#P35G-1.5-14-C, MatTek Corp, Ashland, MA). After 24 hours, media was replaced with either standard media, media with DMSO vehicle (#D8418, Sigma), media with 10 μM FX11 (#427218, EMD Millipore, Billerica, MA) in 1% DMSO, or media with 50 mM DCA (#347795, Sigma). All cell culture media formulations contained glucose and glutamine. 48 hours after this treatment for FX11 and DCA, cells were imaged at 3–4 different locations in each dish for a total of 100–550 cells imaged per treatment group. NAD(P)H images were first acquired, followed immediately by an FAD image of the same field of view. All imaging experiments were performed after cells equilibrated to room temperature. To ensure cell viability and minimize time spent outside of the cell incubator, experiments were kept brief (<20 minutes). Cell viability was also confirmed following imaging. All cell metabolic inhibitor experiments were repeated in triplicate.

### Enzyme activity assays

LDH (#MAK066) and PDH (#MAK183) Activity Assay Kits (Sigma) were used to quantify enzyme activities in MCF10A and HPDE6 cells. Enzymes were isolated from 1 × 10^6^ cells for each activity assay. Enzyme activity levels are reported in milliUnits, which corresponds to nanomoles of reaction product generated per minute. All enzyme activity experiments were repeated 3–5 times.

### Cellular image analysis

NAD(P)H fluorescence lifetime images of cells were analyzed similarly to images of NADH-enzyme solutions using SPCImage software (Becker & Hickl). Binning of only 3 × 3 pixels was used to preserve spatial resolution (SNR > 15). Equation 3 was used to extract the free and protein-bound configurations of NAD(P)H from decay curves. The lifetime of free NAD(P)H was not fixed in this analysis due to the heterogeneity of the intracellular environment in terms of pH, O_2_, and viscosity. Again, a two-exponential model was chosen because the chi-squared goodness of fit value did not improve for a three-exponential model versus a two-exponential model. NAD(P)H ${\tau }_{m}$ is calculated by taking a weighted average of the free and protein-bound lifetimes:

5 $${\tau }_{m}={\alpha }_{1}\ast {\tau }_{1}+{\alpha }_{2}\ast {\tau }_{2}$$

An automated cell segmentation routine was written using CellProfiler to identify individual cells and extract average fluorescence intensity and fluorescence lifetime values for each cell in the field of view (minus background and nuclear signals)^61^. Intensity values for each pixel were calculated by integrating the decay curve corresponding to that pixel. Optical redox ratio values were calculated for each pixel (without binning) by dividing the intensity of NAD(P)H by the intensity of FAD. Then, the average redox ratio across all pixels in each cell cytoplasm was calculated. The redox ratio on a per-cell basis was used for all statistical comparisons. Reported mean redox ratios are normalized to control values. The mean lifetime (${\tau }_{m})\,$of NAD(P)H, which represents the weighted average of the free and protein-bound lifetimes, was calculated for each cell cytoplasm using Equation 5. For each imaging variable, values for all cells in a dish were averaged together. An analysis of the sources of variability in NAD(P)H FLIM signals in our cell experiments shows that error due to fitting of decay curves is far outweighed by biological variation (Supplementary Table 1).

### Cellular fuel sources

1 × 10^5^ MCF10A cells were seeded 24 hours prior to starvation in 35 mm glass-bottom dishes. Then, standard media was replaced with glucose-free, pyruvate-free, and serum-free DMEM (#11966, Gibco) overnight. FLIM images of NAD(P)H in three fields of view were taken in each dish immediately before and immediately after the replacement of media with fresh serum-free media containing either 10 mM sodium pyruvate or 10 mM sodium lactate (#P2256 and #L7022, Sigma). A total of 200–300 cells were imaged per condition, and individual cells were analyzed. This experiment was repeated in triplicate.

### Statistical analysis

Differences in optical redox ratios, NAD(P)H intensities, and FAD intensities between treatment groups in cells were tested across 3 or more replicates using a ratio paired student’s t-test. All standard deviations in figures are calculated across experimental replicates. Differences in NAD(P)H lifetime variables between solutions and differences in cellular NAD(P)H lifetime variables between treatment groups were tested across 3 or more replicates using an unpaired student’s t-test with Welch’s correction for samples of unequal variance. Differences in enzyme activity levels between control and treated cells were tested across 3–5 experiments using the same test. A significance level of 0.05 was used to determine statistical significance in all tests.

### Data availability

The data that support the findings of this study are available from the corresponding author upon reasonable request.

## Electronic supplementary material

Supplementary Information
