# Supplementary material for: Protein-bound NAD(P)H Lifetime is Sensitive to Multiple Fates of Glucose Carbon
Source: Sci Rep. 2018 Apr 3;8:5456. doi: 10.1038/s41598-018-23691-x (PMC5883019; doi:10.1038/s41598-018-23691-x)
Supplement: Supplementary file 1 — Supplementary Information [file 41598_2018_23691_MOESM1_ESM.pdf]

Supplementary Information

Protein-bound NAD(P)H Lifetime is Sensitive to Multiple Fates of Glucose Carbon

Joe T. Sharick[1,2], Peter F. Favreau[2], Amani A. Gillette[2,3], Sophia M. Sdao[4], Matthew J. Merrins[5,6,7], Melissa C. Skala[2,3,*]

[1]Department of Biomedical Engineering, Vanderbilt University, Nashville, Tennessee, USA. [2]Morgridge Institute for Research, Madison, WI, USA. [3]Department of Biomedical Engineering, University of Wisconsin-Madison, Madison, WI, USA. [4]Integrated Program in Biochemistry, University of Wisconsin-Madison, Madison, WI, USA. [5]Department of Medicine, University of Wisconsin-Madison, Madison, WI, USA. [6]William S. Middleton Memorial Veterans Hospital, Madison, WI, USA. [7]Department of Biomolecular Chemistry, University of Wisconsin-Madison, Madison, WI, USA.

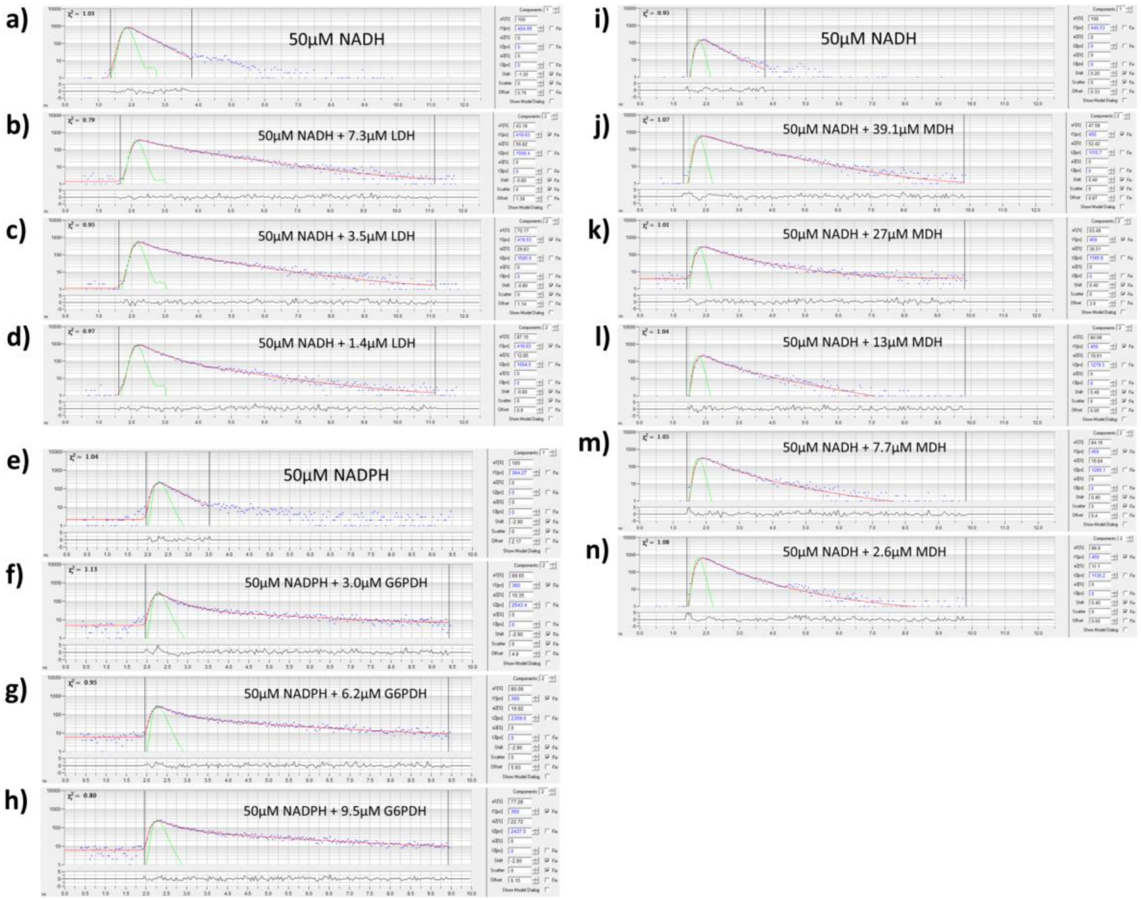

Supplementary Figure 1. NAD(P)H fluorescence lifetime decay curve and fitting examples from single-enzyme binding experiments in solution. (A-D) NADH fluorescence lifetime decay curves from a representative 1x1 binned pixel of solutions of 50 µM NADH alone or mixed with the indicated concentration of LDH. (E-H) NADPH fluorescence lifetime decay curves from a representative 1x1 binned pixel of solutions of 50 µM NADPH alone or mixed with the indicated concentration of G6PDH. (I-N) NADH fluorescence lifetime decay curves from a representative 1x1 binned pixel of solutions of 50 µM NADH alone or mixed with the indicated concentration of MDH (experiments from Fig. 1). Screengrabs taken from SPCImage software (Becker & Hickl) (4). Blue dots represent lifetime decay histogram data (y-axis = number of photons, logarithmic scale). Green line represents the instrument response function (IRF). Red line indicates exponential decay fit. t1, t2, a1, and a2, refer to τ_1, τ_2, α_1, and α_2 respectively (Equation 3). The "components" box determines whether decay curves are fit using the sum of 1, 2, or 3 exponential decays. The "shift" box gives the number of time channels between the IRF

and rising edge of the fluorescence curve. The “scatter” box represents the contribution of signal from scattering photons, which is set to zero in these experiments. The “offset” box (in number of photons) represents the constant baseline level of light (parameter C in Eq. 3), and is calculated from time channels before the rising part of the fluorescence decay. The χ^2 goodness of fit values are close to 1 in all experiments, indicating excellent fits to the lifetime decay curves. Deviations between fit and photon count data are represented by a weighted residual plot below the decay plot.

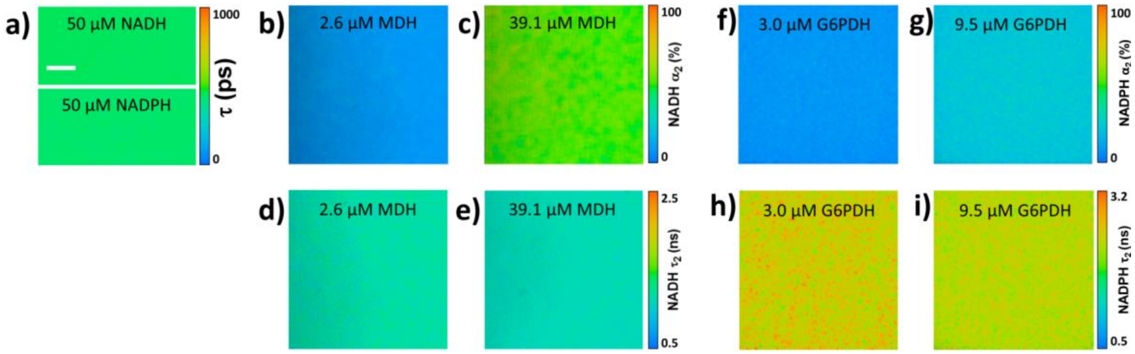

Supplementary Figure 2. NAD(P)H fluorescence lifetime example images from enzyme mixture binding experiments in solution. (**a**) Fluorescence lifetime images of 50 μM NADH and 50 μM NADPH. (**b,c**) Representative images, color-coded for α_2, of mixtures containing 50 μM NADH and 2.6 μM MDH (**b**), and 50 μM NADH and 39.1 μM MDH (**c**). (**d,e**) Representative images, color-coded for τ_2, of NADH-MDH mixtures containing 50 μM NADH and 2.6 μM MDH (**d**), and 50 μM NADH and 39.1 μM MDH (**e**). (**f,g**) Representative images, color-coded for α_2, of NADPH-G6PDH mixtures containing 50 μM NADPH and 6.0 μM G6PDH (**f**), and 50 μM NADPH and 18.9 μM G6PDH (**g**). (**h,i**) Representative images, color-coded for τ_2, of NADPH-G6PDH mixtures containing 50 μM NADPH and 6.0 μM G6PDH (**h**), and 50 μM NADPH and 18.9 μM G6PDH (**i**). Scale bar = 50 μm. All images binned 5x5 pixels for clarity.

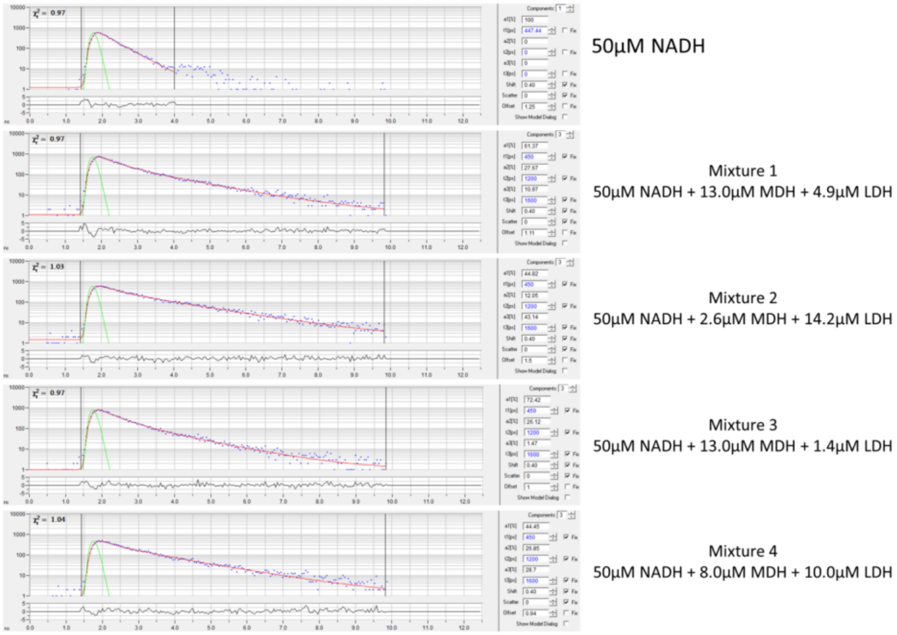

Supplementary Figure 3. NADH fluorescence lifetime decay curve and fitting examples from enzyme mixture binding experiments in solution. NADH fluorescence lifetime decay curves from a representative 1x1 binned pixel of 50 µM NADH alone or mixed with the indicated concentrations of LDH and MDH (experiments in Fig. 2). See Supplemental Figure 1 legend for explanation of SPCImage parameters. t1-3 refer to τ_1, τ_2, and τ_3 respectively and are fixed according to Equation 4. a1-3 refer to $\alpha_{NADH-Free}$, $\alpha_{NADH-MDH}$, and $\alpha_{NADH-LDH}$ respectively (Eq. 4).

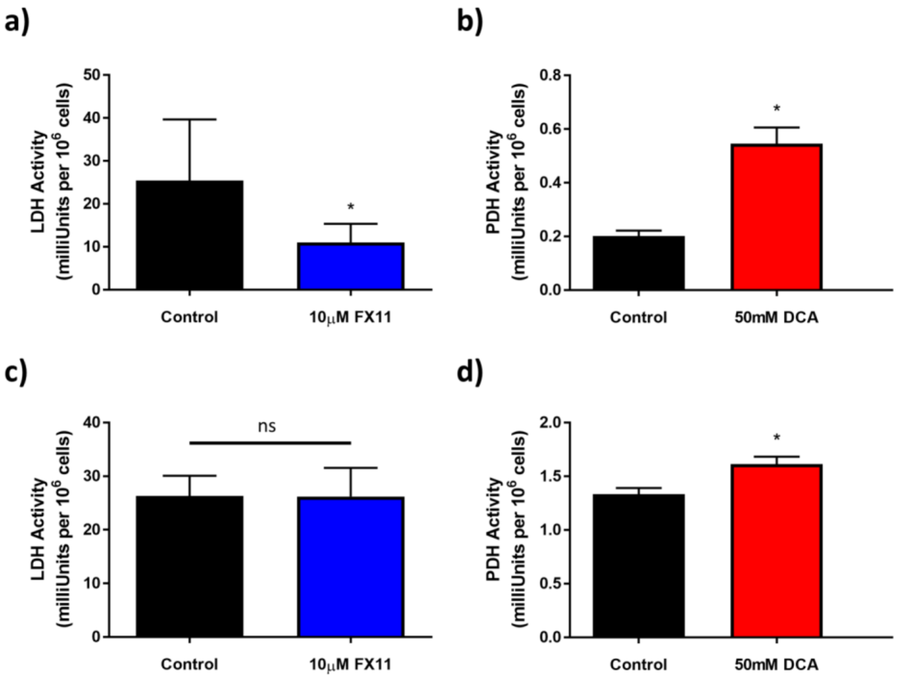

Supplementary Figure 4. Absolute activities of PDH and MDH in cells. (A-B) Mean and standard deviations of the absolute LDH (A) and PDH (B) activities in 10^6 MCF10A cells after 48 hours of 10μM FX11 and 48 hours of 50mM DCA treatment vs. vehicle, respectively. * $p<0.05$ vs. control. n = 3-4 experiments. (C-D) Mean and standard deviations of the absolute LDH (C) and PDH (D) activities in 10^6 HPDE6 cells after 48 hours of 10μM FX11 and 48 hours of 50mM DCA treatment vs. vehicle, respectively. n = 3-5 experiments.

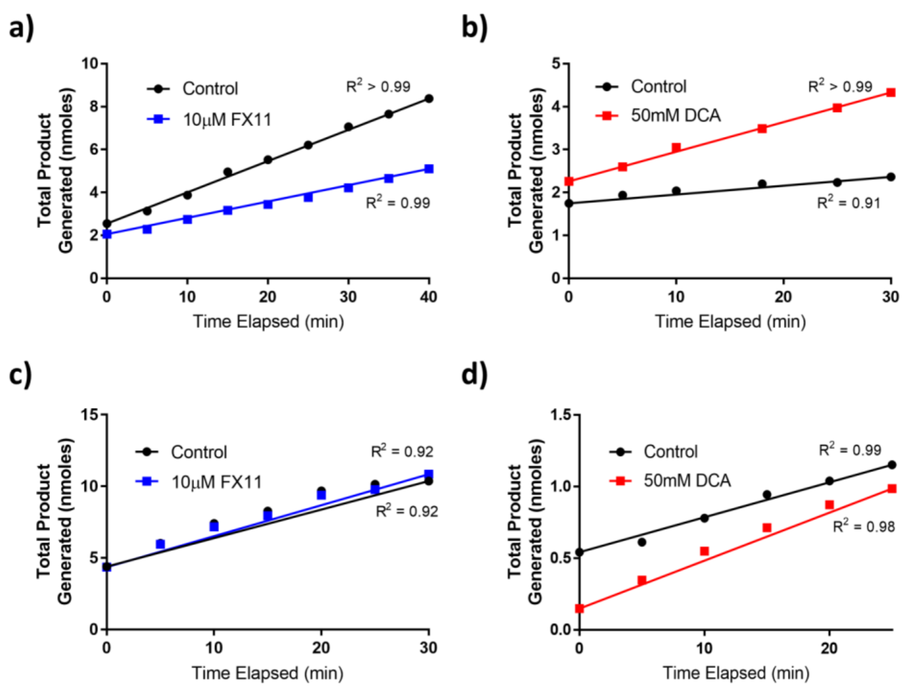

Supplementary Figure 5. Examples of time-dependent LDH and PDH activity with fitting. (A-B) NADH generated over time by LDH (A) and PDH (B) isolated from 10^6 MCF10A cells after 48 hours of 10µM FX11 and 48 hours of 50mM DCA treatment vs. vehicle, respectively. Example data is from one single reaction from one experiment. Linear fits are calculated using first and last points in each reaction. The coefficient of determination (R^2) accounts for all time points. (C-D) NADH generated over time by LDH (C) and PDH (D) isolated from 10^6 HPDE6 cells after 48 hours of 10µM FX11 and 48 hours of 50mM DCA treatment vs. vehicle, respectively. Example data shown from one single reaction per line.

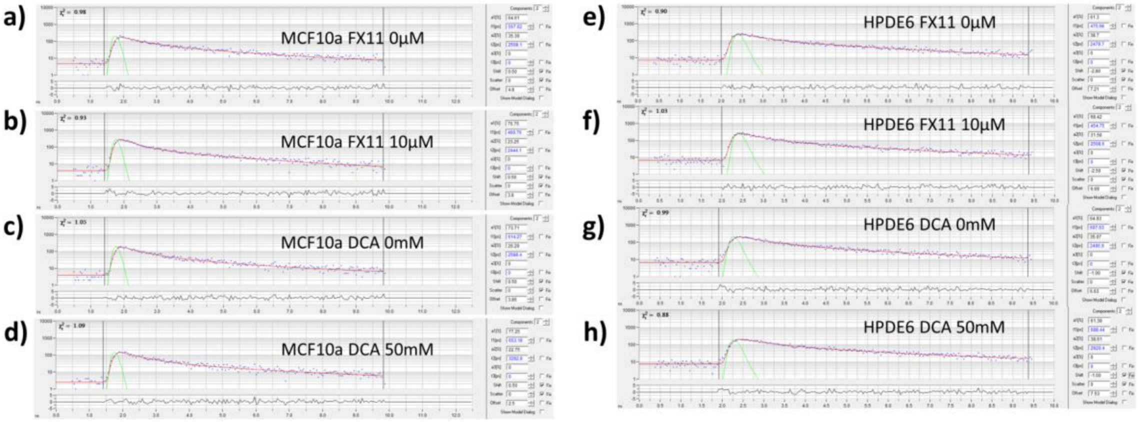

Supplementary Figure 6. NAD(P)H fluorescence lifetime decay curve and fitting examples from MCF10A and HPDE6 cells. (A-D) NAD(P)H fluorescence lifetime decay curves from a representative 1x1 binned pixel in the cytoplasm of MCF10A cells under each of the indicated metabolic inhibitor treatment conditions. (E-H) NAD(P)H fluorescence lifetime decay curves from a representative 1x1 binned pixel in the cytoplasm of HPDE6 cells under each of the indicated metabolic inhibitor treatment conditions (experiments in Fig. 3-9). See Supplemental Figure 1 legend for explanation of SPCImage parameters.

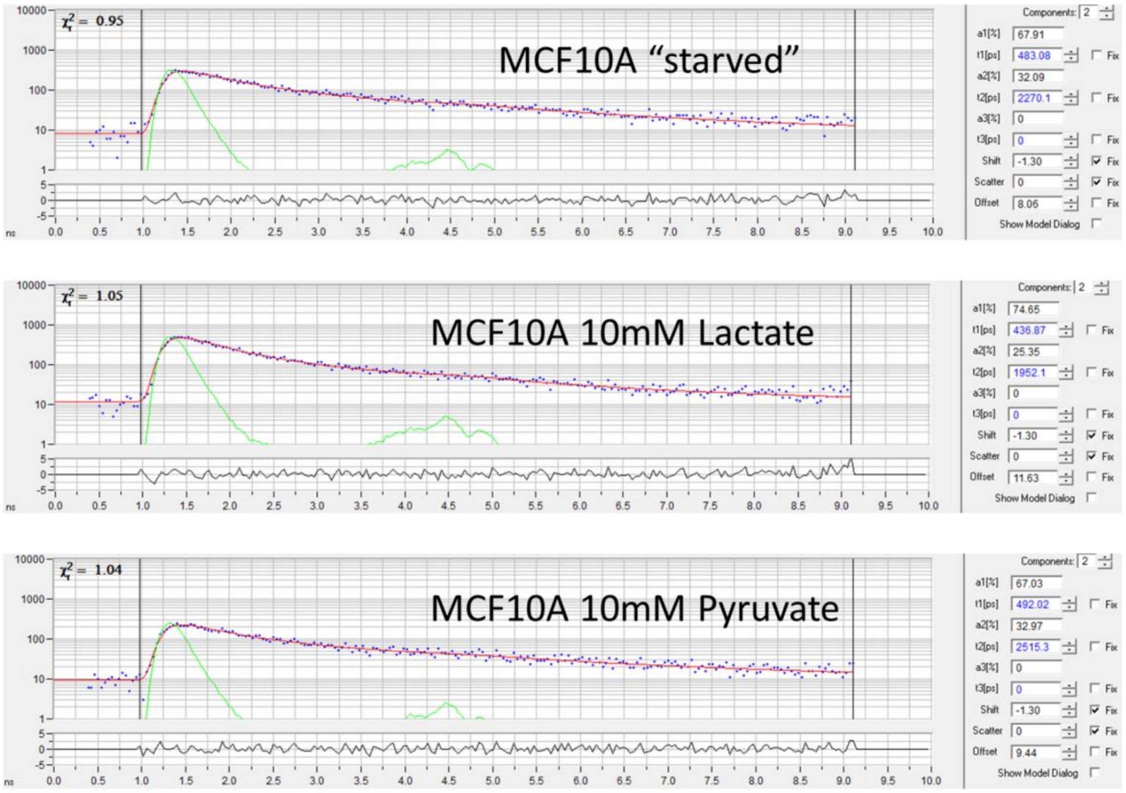

Supplementary Figure 7. NAD(P)H fluorescence lifetime decay curve and fitting examples from starved MCF10A fed with pyruvate or lactate. NAD(P)H fluorescence lifetime decay curves from a representative 1x1 binned pixel in the cytoplasm of MCF10A cells that have been starved or fed the indicated fuel source (experiments in Fig. 10). See Supplemental Figure 1 legend for explanation of SPCImage parameters.

		MCF10A				HPDE6			
		FX11 Ctrl	FX11 10µM	DCA Ctrl	DCA 50mM	FX11 Ctrl	FX11 10µM	DCA Ctrl	DCA 50mM
NAD(P)H τ_2 Coefficient of Variation (%)	Experimental Replicates	1.3	0.9	0.2	0.5	1.9	1.3	0.7	0.5
	Cell	3.6	2.9	3.1	4.4	2.4	2.3	2.8	2.8
	Pixel	13.0	16.0	18.1	16.8	8.2	8.5	10.8	11.2
	Within a pixel	2.0	2.0	2.2	2.4	2.0	2.0	2.2	2.2
	**Proportion due to uncertainty within a pixel (%)**	**2.1**	**1.5**	**1.4**	**1.9**	**5.0**	**4.9**	**3.6**	**3.5**
NAD(P)H τ_1 Coefficient of Variation (%)	Experimental Replicates	5.0	2.4	3.1	4.2	3.7	5.1	7.4	8.1
	Cell	5.8	5.7	5.2	6.6	3.1	3.6	4.1	5.5
	Pixel	20.7	29.6	24.8	25.5	11.5	14.1	16.0	16.4
	Within a pixel	4.4	3.5	4.4	4.4	4.4	4.4	4.5	5.6
	**Proportion due to uncertainty within a pixel (%)**	**3.8**	**1.3**	**2.9**	**2.6**	**10.9**	**7.6**	**5.8**	**7.8**
NAD(P)H α_1 Coefficient of Variation (%)	Experimental Replicates	8.6	1.8	6.7	4.0	0.9	2.1	2.5	2.5
	Cell	2.9	1.7	2.2	2.5	3.5	2.9	3.0	4.9
	Pixel	9.4	5.6	9.0	8.8	4.8	7.7	9.8	10.3
	Within a pixel	1.7	1.2	1.8	2.0	1.8	1.9	2.1	2.9
	**Proportion due to uncertainty within a pixel (%)**	**1.7**	**3.7**	**2.4**	**3.9**	**8.2**	**4.5**	**3.7**	**5.8**

Supplementary Table 1. Sources of variability in NAD(P)H FLIM measurements. Coefficients of variation and proportion of variation due to fitting uncertainty for NAD(P)H τ_2, τ_1, and α_1 for each treatment condition in both cell types. The variation of NAD(P)H τ_1, τ_2, and α_1 in each cell/treatment group within an individual pixel due to fitting was calculated by simulating decay curves with τ_1, τ_2, and α_1 values that represented the measured values in each cell/treatment group (Fig. 7-9). For each of the 8 conditions, 65,536 curves were simulated using MATLAB (MathWorks) and random Poisson noise (square root of the number of photons) was added to each curve. The number of photons and the SNR of the simulated curves represent those acquired in our cell experiments. Each curve was fit in SPCImage (Becker & Hickl) and the standard deviation of the new fitted parameters was used to determine the coefficient of variation of fitting within a pixel. Coefficients of variation between experimental replicates, between cells within an experiment, and between pixels within a single cell (all factors that make up biological variation) were also quantified for each condition by calculating the coefficient of variation (ratio of standard deviation to mean). The proportion of total variance (sum of squared coefficients of

variance) of a lifetime parameter that can be attributed to variability within a pixel (squared coefficient of variance within a pixel) was calculated and is displayed for each treatment condition and cell type.
